# Preoperative Diagnosis and Treatment Outcomes of Incarcerated Inferiorly Displaced Flap Tear of the Medial Meniscus: Comparison between Flap Tears with and without Incarcerated Fragment

**DOI:** 10.1155/2018/5941057

**Published:** 2018-05-23

**Authors:** Min Jung, Dong Hoon Lee, Sung-Jae Kim, Chong Hyuk Choi, Sung-Hwan Kim, Seokhwan Jin, Kun-Bo Park

**Affiliations:** ^1^Arthroscopy and Joint Research Institute, Department of Orthopaedic Surgery, Yonsei University College of Medicine, Seoul, Republic of Korea; ^2^Department of Orthopaedic Surgery, Yonsei University College of Medicine, Seoul, Republic of Korea

## Abstract

The purpose of this study was to compare preoperative variables and postoperative outcomes between flap tears with and without incarceration of inferiorly displaced fragments of medial meniscus and find distinct features of incarcerated flap tear of medial meniscus to improve preoperative diagnosis. 79 patients who underwent partial meniscectomy for flap tear of medial meniscus were classified into two groups: group U, usual flap tear without incarcerated fragment; group I, flap tear with incarcerated inferiorly displaced fragment. Patient characteristics and preoperative variables including duration of symptom aggravation were investigated. A comprehensive physical examination including joint line tenderness was performed. Magnetic resonance imaging (MRI) examination was carried out on all patients. Clinical assessments were performed with functional scores including visual analogue scale (VAS), and radiologic evaluation was conducted. Preoperative values and postoperative outcomes measured at the minimum follow-up duration of 2 years were compared between the groups. The groups did not differ significantly regarding postoperative outcomes by functional and radiological evaluations (*p* > 0.05). In making preoperative diagnosis, sensitivity of diagnosis based solely on MR images was significantly lower in group I (68.8%) than that in group U (90.5%) (*p* = 0.040). The following clinical features differed significantly between the groups: Patients in group I had higher scores in preoperative VAS (group U = 6.6; group I = 7.7) (*p* = 0.011) and shorter duration of symptom aggravation (group U = 13.8 weeks; group I = 3.9 weeks) (*p* < 0.001). Joint line tenderness was positive more distinctly in group I (100%) than in group U (74.6%). If displaced flap tear was properly resected, improved outcomes did not differ regardless of incarceration of flap tear. In diagnosing incarcerated inferiorly displaced flap tear, sensitivity of diagnosis based solely on MR images could be low. Distinguishing clinical findings would be helpful in obtaining a more appropriate diagnosis.

## 1. Introduction

Meniscus tear is one of the most common injuries of the knee. The annual incidence of meniscectomy was reported to be 60~70 cases per 100,000 population [1]. In treating meniscus tear, if symptoms persist with conservative treatment, surgical treatment could be performed. Among a variety of tear patterns, flap tear with displaced meniscal fragment tends to be refractory to conservative managements and is more likely to lead to operation of meniscectomy because of the persistent pain and mechanical symptoms. The principles for meniscectomy include removing all unstable fragments and leaving no sudden changes in the contour of meniscal rim [2]. In order to adhere to this principle and treat meniscus tear appropriately, the accurate diagnosis before and during operation has an important significance. However, in case of horizontal flap tear [3], when the displaced fragment of inverted inferior leaf is located inferior and medial to the tibial plateau and incarcerated between the deep fibers of the medial collateral ligament and the tibia, diagnosis can be difficult because it looks as if there is no tear on the surface of the meniscus. For such a reason, it can escape detection during arthroscopic examination and proper treatment cannot be performed, although incarcerated fragment of inferiorly displaced flap tear is the main cause of the symptom. Therefore, it is of great importance to recognize this lesion before surgery. In terms of preoperative diagnosis of inferiorly displaced flap tear of medial meniscus, previous studies have reported radiologic findings [4, 5] including magnetic resonance imaging (MRI) and ultrasonography and examination maneuver [6]. However, accuracy of diagnosis based solely on radiologic finding was noted to be low [6], and it still needs more elements to compensate for deficiency in diagnosing incarcerated inferiorly displaced flap tear of medial meniscus. To the best of our knowledge, there has been no previous study on overall clinical manifestations for preoperative diagnosis and treatment outcomes of this type of meniscal lesion. Accordingly, the purpose of the present study was to compare preoperative variables and postoperative outcomes between flap tears with and without incarceration of inferiorly displaced fragments of medial meniscus and find the distinguishable clinical features of incarcerated flap tear of medial meniscus to improve preoperative diagnosis. It was hypothesized that postoperative outcomes would not differ regardless of incarceration of flap tear if displaced flap tear is properly resected, and there would be distinguishing clinical features to obtain diagnosis with increase in accuracy for incarcerated inferiorly displaced flap tear of medial meniscus.

## 2. Materials and Methods

### 2.1. Patients

**Figure 1:**
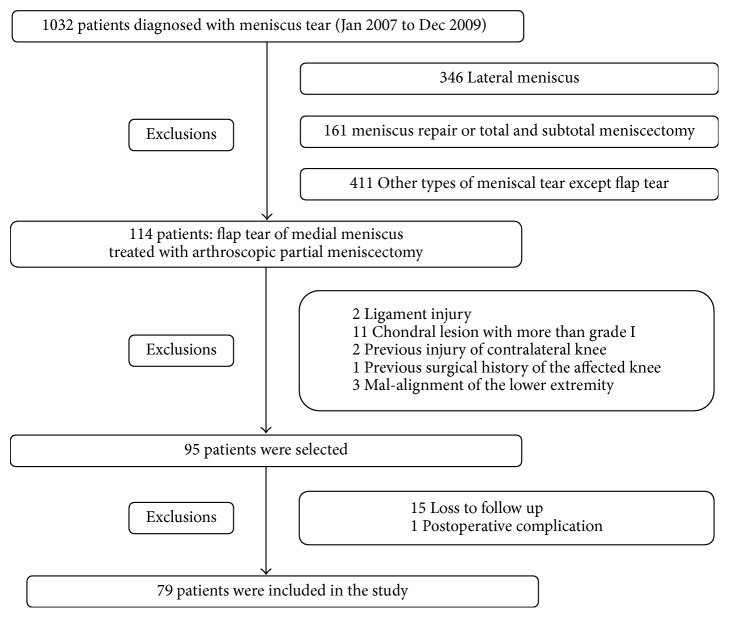
Flowchart of patient inclusion in the study.

A total of 1032 patients who were diagnosed with meniscal tear from January 2007 to December 2009 at the institution were retrospectively reviewed after approval of the study by the institutional review board. Patients were included in the present study according to the following inclusion criteria: (1) isolated medial meniscus lesion arthroscopically diagnosed with flap tear; (2) meniscal tear treated with arthroscopic partial meniscectomy [7]; (3) no arthritic change on preoperative plain radiographs (International Knee Documentation Committee [IKDC] radiologic grade [8] of normal); and (4) a minimum follow-up duration of two years. The exclusion criteria were as follows: (1) meniscus tear treated with repair; (2) lateral meniscus tear; (3) concomitant chondral lesion of higher than grade I according to the Outerbridge grading system at arthroscopy [9]; (4) concomitant ligament injury; (5) cystic lesion; (6) Bony deformity of femur or tibia; (7) malalignment of the lower extremity (the normal mechanical axis line passes a mean distance [and standard deviation] of 8 ± 7 mm medial to the center of the knee joint line on standing hip-knee-ankle radiographs [10]); (8) previous surgery of the affected knee; (9) previous injury of the contralateral knee; and (10) postoperative complication. Fifteen patients who met the inclusion criteria were excluded because of loss to follow-up. According to the selection process of included patients by inclusion and exclusion criteria, seventy-nine patients were included in the current study (Figure 1).

**Figure 2:**
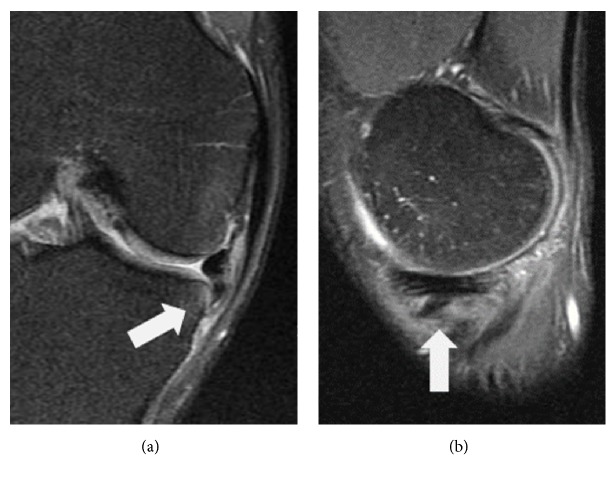
MRI of a 33-year-old male patient with an incarcerated inferiorly displaced flap tear of medial meniscus. (a) A coronal T2-weighted image and (b) a sagittal T2-weighted image of the most medial aspect of the knee showed that the displaced fragment (arrow) was located inferomedial to the tibial plateau and extended deep between tibia and medial collateral ligament.

**Figure 3:**
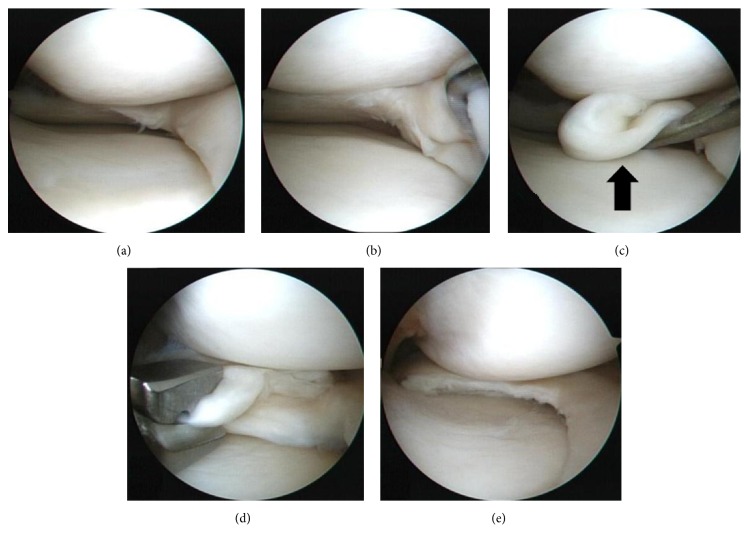
Arthroscopic findings of the same patient as Figure 2 with an inferiorly displaced flap tear of medial meniscus. (a) The incarcerated meniscal fragment was not seen before exploration. (b & c) The large torn fragment (arrow) was found when the medial meniscus was elevated with a probe. (d) The fragment was resected with an arthroscopic basket punch. (e) Arthroscopic partial meniscectomy was performed.

The included patients were divided into two groups according to whether or not meniscal lesion had an incarceration of displaced fragment of flap tear. Group U included sixty-three patients who underwent partial meniscectomy for usual flap tear of medial meniscus without incarceration of displaced fragment. In the present study, usual flap tear represented vertical flap tear or horizontal flap tear without incarceration according to the ISAKOS classification [3]. Group I included sixteen patients who underwent partial meniscectomy for flap tear with incarcerated fragment of inverted inferior leaf (Figure 2). All surgeries were performed by a single surgeon, the senior author. After comprehensive exploration of the entire knee joint initially, careful probing was performed particularly in the areas of meniscal tear. In patients of suspicion of incarcerated flap tear preoperatively, inferior recess below the medial meniscus around the suspected area was examined thoroughly with probe. After pattern of meniscus tear and location were confirmed, resection of the unstable portion of the torn meniscus was performed (Figure 3). After completing meniscectomy, the width of remaining meniscus was measured with a graduated probe (Video clip). On the basis of previous studies, a procedure that left a width of remaining meniscus more than 5 mm was defined as partial meniscectomy [11]. Only patients who underwent partial meniscectomy were included in the present study.

### 2.2. Preoperative Magnetic Resonance Imaging (MRI) Examination

Preoperatively, MRI examination was performed in all patients. MRI scanner (Magnetom Vision and Sonata, Siemens Medical System, Erlangen, Germany) included two 1.5 tesla superconducting magnets using quadrature extremity coils. The MR protocol incorporated the following sequences: fat-suppressed intermediate weighted images in the axial plane (TR [repetition time] = 3600 ms, TE [echo time] = 90 ms, FOV [field of view] = 150 mm × 150 mm, slice thickness = 4 mm), T1-weighted images in the sagittal plane (TR = 520 ms, TE = 14 ms, FOV = 160 mm × 160 mm, slice thickness = 4 mm), fat-suppressed dual-echo T2-weighted images in the sagittal plane (TR = 2700 ms, TE = 30 ms, FOV = 160 mm × 160 mm, slice thickness = 2 mm), and dual-echo T2 weighted images in the coronal plane (TR = 3700 ms, TE = 90 ms, FOV = 160 mm × 160 mm, slice thickness = 3 mm). The MR images were reviewed and interpreted by an experienced musculoskeletal radiologist, using the Picture Archiving and Communication System (PACS) workstations (Marosis, Infiniti, Seoul, Republic of Korea). After operation, the MR images were reviewed again by orthopaedic surgeons and musculoskeletal radiologist. To ensure a consistency in the opinions of these doctors, a conclusion about postoperative reading of MR images was drawn after discussion at the conference where the orthopaedic surgeons and musculoskeletal radiologist were present together. Incarcerated inferiorly displaced flap tear of medial meniscus could be diagnosed when the displaced fragment of flap tear which was located inferomedial to the tibial plateau and extended deep between tibia and medial collateral ligament was found on MR images (Figure 2).

### 2.3. Clinical Assessments

Patient characteristics, preoperative variables, and follow-up outcomes measured at the minimum follow-up duration of 2 years after operation were assessed. Before the operation, all patients were asked to complete a standardized questionnaire including demographic information, trauma history, and total duration and recent aggravation period of symptoms. Demographic data for patients included sex, age, body mass index (BMI), and affected side. A comprehensive physical examination including joint line tenderness, maximal tender point, and McMurray test was performed and the findings were recorded on a data collection sheet. After operation, arthroscopic findings including pattern and location of meniscal tear were recorded on the operation note. Locations of tear were divided into following categories: anterior horn, body, posterior horn, and more than one portion.

All patients were evaluated in regard to clinical function and radiologic findings preoperatively and postoperatively. Postoperative follow-up evaluations were performed at 6 months and annually during a patient's regular annual visit. Clinical function was assessed with a ten-point visual analogue scale (VAS), the Lysholm knee scoring scale [12], the International Knee Documentation Committee (IKDC) subjective knee evaluation form [13], and the Tapper and Hoover grading system [14]. VAS is a measure of pain intensity. It is a continuous scale comprised of a horizontal visual analogue scale. The scale consists of “no pain” (score of 0) to “worst imaginable pain” (score of 10) on 10-mm scale. Patients were asked to report current pain intensity. The Lysholm knee scoring scale [12] consists of eight items that measure limp (5 points), need for support (5 points), locking sensation (15 points), giving way sensation (25 points), pain (25 points), swelling (10 points), climbing stairs (10 points), and squatting (5 points). The total score is made up of the sum of the individual responses to each of the eight questions, with a perfect score of 100. Higher scores mean a better condition of knee with fewer disability or symptoms. The postoperative Lysholm knee scoring scale was also classified into the following four grades according to Mitsou et al. [15]: excellent = 95–100; good = 84–94; fair = 65–83; poor = <65. The IKDC subjective knee evaluation form [13] consisted of three categories: symptoms, sports activities, and function. The patient's responses to each question are scored using an ordinal method such that a score of 0 is given to responses that represent the highest level of symptoms or lowest level of function. Higher scores represent lower levels of symptoms and higher levels of function. A score of 100 is interpreted to indicate the absence of symptoms and no limitation with activities of daily living or sports activities. The postoperative IKDC score was also classified into the following four grades by Haas et al. [16]: excellent = 90–100; good = 80–89; fair = 70–79; poor = <70. Patients were assigned one of four grades postoperatively depending on subjective symptoms and disability according to the Tapper and Hoover grading system [14], which are composed of the following grades: excellent: the patient had no symptoms and no disability related to the knee; good: the patient had minimal symptoms, such as aching or weakness after heavy use or effusion after heavy exertion, but there was essentially no disability; fair: the patient had symptoms such as trouble kneeling or climbing stairs; weakness, pain, or discomfort had become enough of a problem to interfere somewhat with everyday activities, and the patient thought he or she had some disability; the patient was active but could not participate in vigorous sports (such as skiing, tennis, and football); poor: the symptoms were severe and included all of those listed under fair as well as the presence of pain at rest, limited motion, and locking; the patient was clearly disabled, and his or her activities, including walking, were definitely limited because of his or her knee.

For radiological evaluation, radiographs of anteroposterior view, lateral view, and 45° flexion weightbearing posteroanterior view and Merchant view were obtained preoperatively and annually after operation. Interpretation of radiographs was made by two trained clinical fellows blinded to the diagnosis of patients to reduce the effect by subjectivity. To ensure consistency in the opinions of the two doctors, the interpretation of radiographs was discussed at the same table by two doctors and a conclusion on the interpretation was drawn. Patients were graded as follows according to the IKDC radiographic assessment scale [8]: A = normal; B = near-normal, greater than 4 mm joint space but early osteophyte; C = abnormal, joint space 2 to 4 mm, or greater than 50%; D = severely abnormal, joint space less than 2 mm, or less than 50%.

### 2.4. Statistical Analysis

Continuous variables were tested for normality using Shapiro–Wilk test. To compare the groups in terms of patient characteristics and preoperative and postoperative values, the independent-samples *t*-test or the Mann–Whitney *U* test was employed for continuous variables and the chi-square test or the Fisher exact test was used for categorical variables. Comparison between preoperative and postoperative values regarding VAS score, the Lysholm knee score, and the IKDC subjective score was performed using the paired *t*-test or the Wilcoxon signed rank test. Statistical analysis was performed with SPSS software (version 23.0; IBM), and *p* < 0.05 was considered significant. Statistical power was calculated using G^*∗*^Power (version 3.1) [17].

## 3. Results

There were 26 male and 37 female patients in group U and 7 male and 9 female patients in group I. The average age at the time of surgery was 42.7 years (range, 21–59 years) in group U and 45.6 years (range, 33–65 years) in group I. The mean BMI was 24.8 kg/m^2^ (range, 20.7–27.8 kg/m^2^) in group U and 23.7 kg/m^2^ (range, 21.6–26.9 kg/m^2^) in group I. Affected side was as follows: right = 39 cases and left = 24 cases in group U; right = 10 cases and left = 6 cases in group I. Proportion of patients with history of trauma was 52.4% (33 cases) in group U and 37.5% (6 cases) in group I. The mean of total duration of symptom was 24.7 months (range, 1–132 months) in group U and 18.3 months (range, 3–84 months) in group I. There was no statistically significant difference between the groups regarding sex, mean age, mean BMI, affected side, mean duration of symptoms before operative treatment, or proportion of patients with history of trauma (*p* > 0.05). The groups differed significantly with respect to the mean duration of aggravation of symptom: 13.8 weeks (range, 2–52 months) in group U; 3.9 weeks (range, 1–8 weeks) in group I (*p* < 0.001). Among patients in group I, twelve patients (75%) had operation with recent aggravating pain within one month preoperatively. There was no statistically significant difference in location of tear between the groups (*p* = 0.678). The groups did not differ significantly in regard to the mean of the preoperative Lysholm knee score (*p* = 0.098) and the mean of the IKDC subjective score (*p* = 0.605). However, there was a statistically significant difference in the mean of the preoperative VAS score: group U = 6.6; group I = 7.7 (*p* = 0.011) (Table 1). The statistical power assessed using G^*∗*^Power [17] was 74.0% with regard to VAS score. No patient included in the present study underwent a revision operation for meniscal retear.

Comparison between the preoperative values and the postoperative values measured at the minimum follow-up duration of 2 years within each group was performed. Mean duration of follow-up was 26.4 months in group U and 27.2 months in group I. The groups had statistically significant differences between preoperative and postoperative values for the VAS score (*p* < 0.001), Lysholm knee score (*p* < 0.001), and the IKDC subjective score (*p* < 0.001) (Table 2).

Postoperative functional and radiologic outcomes were compared between the groups. The mean postoperative VAS score was 1.6 in group U and 1.3 in group I, the mean postoperative Lysholm knee score was 95.0 in group U and 93.4 in group I, and the mean postoperative IKDC subjective score was 92.1 in group U and 91.7 in group I. These functional scores did not show statistically significant differences between the groups (*p* > 0.05). The grades assigned according to the Lysholm knee score, the IKDC subjective score, and the Tapper and Hoover grading systems also did not show statistically significant differences between the groups (*p* > 0.05). The groups did not differ significantly regarding IKDC radiographic scales (*p* = 0.723) (Table 3).

According to the preoperative physical examination, 74.6% of patients in group U had tenderness on the medial joint line, whereas 100% of patients in group I had tenderness, and the maximal tender point was located just below the joint line in 12 of 16 patients (75%). Proportions of patients who had tenderness differed significantly between the groups (*p* = 0.032). 71.4% of patients in group U and 75.0% of patients in group I tested positive for McMurray test (*p* > 0.999). Sensitivity of the preoperative diagnosis based on MR images was 90.5% in group U and 68.8% in group I. The groups differed significantly (*p* = 0.040). After operation, MR images were reviewed again. According to the postoperative review of MR images with reference to arthroscopic findings, sensitivity of diagnosis for flap tear was improved to 95.2% in group U and 93.8% in group I (*p* > 0.999) (Table 4).

## 4. Discussion

There has been no previous study dealing with overall clinical features including patient characteristics, preoperative factors, radiologic findings, and postoperative outcomes of incarcerated inferiorly displaced flap tear of medial meniscus. The present study focused on comparison of clinical findings between flap tears with and without incarceration of inferiorly displaced fragments of medial meniscus, as well as the distinguishing clinical features of incarcerated flap tear of medial meniscus to improve preoperative diagnosis.

According to the results of the present study, there is no significant difference of postoperative outcomes between the groups, and both groups had significantly improved postoperative outcomes compared to preoperative values. In light of these, if displaced flap tear is properly resected, postoperative outcomes improve regardless of incarceration of displaced flap tear. However, critical point in treating incarcerated inferiorly displaced flap tear of medial meniscus is that appropriate diagnosis should be made preoperatively and intraoperatively. The displaced fragment of inferior leaf in flap tear of the medial meniscus can be located inferior and medial to the tibial plateau and incarcerated between the medial aspect of the tibial plateau and the deep fibers of the medial collateral ligament. In this case, the surface of the meniscus may appear to be free from tear during arthroscopy unless inferior recess below the medial meniscus around the suspected area was examined thoroughly using probe. Accordingly, preoperative proper identification of the incarcerated flap tear can be an essential prerequisite for appropriate treatment by obtaining detection of hidden cause of symptom intraoperatively without mistake.

MR imaging has been known to be a sensitive and noninvasive diagnostic tool for detecting most of meniscus tears. MR imaging has high sensitivity and specificity for meniscal tears ranging from 90 to 95% [18]. However, sensitivity and specificity of MRI were reported to be, respectively, 71% and 98% for detection of tears with recess fragments [19]. According to a previous radiologic study [4] describing MRI features of inferiorly displaced flap tear, only eight of eleven patients (72.7%) had precise diagnosis for displaced fragment of meniscal flap tear before operation. The present study also showed that eleven of sixteen patients (68.8%) had precise diagnosis based on reading of preoperative MR images by an experienced musculoskeletal radiologist, whereas sensitivity of the preoperative diagnosis for usual flap tear without incarceration based on MR images was fifty-seven of sixty-three patients (90.5%). The poor diagnosis rate according to the MR image was found especially in patients with incarcerated fragments. A previous study [20] noted that sole reliance on MR images without clinical information might lead to inappropriate treatment in 35.1% of patients. The present study also showed that sensitivity of diagnosis for incarcerated inferiorly displaced flap tear based on MR images was increased from 68.8% which was made solely by MR images before surgery to 93.8% which was made by MR images and arthroscopic findings after surgery. As such, MRI alone cannot make accurate diagnoses, and it needs more elements to compensate for deficiency.

The present study demonstrated that patient characteristics and physical examination could make up a weakness of radiological examination in diagnosis for incarcerated inferiorly displaced flap tear of medial meniscus. First, in terms of characteristics of patients about symptom, degree of pain based on preoperative mean VAS score was worse in patients with incarcerated flap tear (VAS score = 7.7) than patients with usual flap tear without incarcerated fragment (VAS score = 6.6) according to the results of the present study (*p* = 0.011). Flap tear has been known to cause persistent pain resulting from mechanical symptoms such as catching and locking [21]. Additionally, pain caused by displaced fragment of flap tear can deteriorate when it becomes incarcerated. Incarcerated fragment of the inferiorly displaced flap tear between the medial collateral ligament and proximal tibia extends deep into the recess [4]. It can be considered to cause traction at the meniscocapsular junction and may be the main cause of acute and deteriorating knee pain including mechanical symptoms. Accordingly, incarcerated flap tear was thought to lead to more aggravating pain than usual flap tear. The present study also showed that mean duration of pain aggravation is shorter in patients with incarcerated flap tear (3.9 weeks) than patients with usual flap tear without incarcerated fragment (13.8 weeks). To sum up, if a patient suspected of having meniscal flap tear presents with recently aggravated severe knee pain within one month, incarcerated flap tear is open to be consideration as a diagnosis.

Second, in terms of findings according to the physical examination, the current study showed that joint line tenderness was positive more distinctly in patients with incarcerated flap tear (100%, 16 of 16 patients) than patients with usual flap tear without incarcerated fragment (74.6%, 47 of 63 patients) (*p* = 0.032). According to the previous studies [22, 23], tenderness over the joint line in patients with medial meniscus tear was reported to be positive in 71% to 88% of the cases depending on patterns of tear and has been noted as the most accurate clinical sign. Patients who had flap tear without incarcerated fragment tested positive in similar proportion to those of a previous study [23], but the positive rate of patients with incarcerated meniscal fragment was higher. Another factor to notice was that the maximal tender point was located just below the joint line in 12 of 16 patients with incarcerated fragments (75%). The accuracy of the clinical diagnosis of meniscus tear can be improved when the results of physical examination are added [6, 24]. The combination of physical findings including high positive rate of tenderness over the joint line and the maximal tender point located just below the joint line would be helpful in obtaining a more appropriate diagnosis for incarcerated inferiorly displaced flap tear.

There were several weaknesses in the current study. First, the study was based on a retrospective review. To draw a solid conclusion, a prospective study is needed. Second, the number of included patients who had incarcerated flap tear was small. The small number of patients could decrease the power for statistical analysis. Third, all the subjects involved in the study were patients who had flap tear with or without incarcerated fragments. Accordingly, only sensitivity of each finding could be obtained and specificity could not be calculated. The accuracy of the test can be determined by both sensitivity and specificity. Further study including both patient group and control group without meniscus lesion is needed. Fourth, the MR images were reviewed and interpreted by an experienced musculoskeletal radiologist with thirteen years of experience. Radiologic assessment inevitably tends to be subjective. Experience is even more important because incarcerated flap tears of the medial meniscus are relatively rare lesion [4]. Accordingly, unavoidable subjectivity regarding radiologic evaluation posed a limitation and could affect the results. In addition, the MR images were reviewed and interpreted by only one radiologist. Research design of MRI diagnosis was weak and could affect the results.

## 5. Conclusions

If displaced flap tear was properly resected, postoperative outcomes were improved and did not differ regardless of incarceration of displaced flap tear. Accurate diagnosis is essential before operation for proper treatment for incarcerated inferiorly displaced flap tears of the medial meniscus. In diagnosing incarcerated inferiorly displaced flap tear, sensitivity of diagnosis based solely on MR images could be low. Distinguishing clinical findings would be helpful in obtaining a more appropriate diagnosis.

## Figures and Tables

**Table 1:** Demographic data for patients and preoperative variables.

Variable	Group U (*n* = 63)	Group I (*n* = 16)	*p* value
Sex^a^			
Male	26 (41.3%)	7 (43.8%)	0.857
Female	37 (58.7%)	9 (56.3%)	
Age (years)^b^	42.7 ± 10.8	45.6 ± 8.3	0.522
BMI (kg/m^2^)^b^	24.8 ± 1.8	23.7 ± 2.0	0.424
Side^a^			
Right	39 (61.9%)	10 (62.5%)	0.965
Left	24 (38.1%)	6 (37.5%)	
Duration of symptom^b^			
Total duration	24.7 ± 33.3	18.3 ± 19.7	0.787
Duration of aggravation	13.8 ± 14.1	3.9 ± 2.1	<0.001
Trauma history^a^			
Yes	33 (52.4%)	6 (37.5%)	0.288
No	30 (47.6%)	10 (62.5%)	
Location of tear^a^			
Posterior horn	21 (33.3%)	4 (25.0%)	0.678
Body	15 (23.8%)	5 (31.3%)	
More than one portion	27 (42.9%)	7 (43.8%)	
VAS score^b^	6.6 ± 1.6	7.7 ± 0.9	0.011
Lysholm knee score^b^	63.6 ± 6.2	61.2 ± 5.2	0.098
IKDC subjective score^b^	59.7 ± 5.8	58.9 ± 5.0	0.605

BMI: body mass index; VAS: visual analogue scale; IKDC: International Knee Documentation Committee. ^a^The values are given as *n* (%). ^b^The values are given as mean ± standard deviation.

**Table 2:** Comparison between preoperative and postoperative values in each group.

Variable	Preoperative value	Postoperative value	*p* value
VAS score^a^			
Group U	6.6 ± 1.6	1.6 ± 1.3	<0.001
Group I	7.7 ± 0.9	1.3 ± 1.2	<0.001
Lysholm knee score^a^			
Group U	63.6 ± 6.2	95.0 ± 4.7	<0.001
Group I	61.2 ± 5.2	93.4 ± 4.7	<0.001
IKDC subjective score^a^			
Group U	59.7 ± 5.8	92.1 ± 6.1	<0.001
Group I	58.9 ± 5.0	91.7 ± 7.4	<0.001

VAS: visual analogue scale; IKDC: International Knee Documentation Committee. ^a^The values are given as mean ± standard deviation.

**Table 3:** Comparison of postoperative variables between the groups.

Variable	Group U (*n* = 63)	Group I (*n* = 16)	*p* value
VAS score^a^	1.6 ± 1.3	1.3 ± 1.2	0.490
Lysholm knee score^a^	95.0 ± 4.7	93.4 ± 4.7	0.121
Lysholm knee score grade^b^			0.653
Excellent	33 (52.4%)	7 (43.8%)	
Good	27 (42.9%)	8 (50.0%)	
Fair	3 (4.8%)	1 (6.3%)	
Poor	0 (0%)	0 (0%)	
IKDC subjective score^a^	92.1 ± 6.1	91.7 ± 7.4	0.932
IKDC subjective score grade^b^			>0.999
Excellent	43 (68.3%)	11 (68.8%)	
Good	14 (22.2%)	4 (25.0%)	
Fair	6 (9.5%)	1 (6.3%)	
Poor	0 (0%)	0 (0%)	
Tapper and Hoover grade^b^			0.894
Excellent	45 (71.4%)	11 (68.8%)	
Good	13 (20.6%)	4 (25.0%)	
Fair	5 (7.9%)	1 (6.3%)	
Poor	0 (0%)	0 (0%)	
IKDC radiographic scale^b^			0.723
A	51 (81.0%)	14 (87.5%)	
B	12 (19.0%)	2 (12.5%)	
C	0 (0%)	0 (0%)	
D	0 (0%)	0 (0%)	

VAS: visual analogue scale; IKDC: International Knee Documentation Committee. ^a^The values are given as mean ± standard deviation. ^b^The values are given as *n* (%).

**Table 4:** Comparison of positive rates of clinical and radiological findings between the groups.

Variable	Group U (*n* = 63)	Group I (*n* = 16)	*p* value
Tenderness^a^			0.032
Yes	47 (74.6%)	16 (100%)	
No	16 (25.4%)	0 (0%)	
McMurray test^a^			>0.999
Yes	45 (71.4%)	12 (75.0%)	
No	18 (28.6%)	4 (25.0%)	
Preoperative MRI diagnosis^a^			0.040
Yes	57 (90.5%)	11 (68.8%)	
No	6 (9.5%)	5 (31.2%)	
Postoperative MRI diagnosis^a^			>0.999
Yes	60 (95.2%)	15 (93.8%)	
No	3 (4.8%)	1 (6.2%)	

MR: magnetic resonance imaging. ^a^The values are given as *n* (%).

## Data Availability

The data used to support the findings of this study are available from the corresponding author upon request.

## Supplementary Materials

Supplementary Materials Video clip: arthroscopic treatment for incarcerated inferiorly displaced flap tear of medial meniscus was performed on 63-year-old female patient.
